# Determinants of Infant Adiposity across the First 6 Months of Life: Evidence from the Baby-bod study

**DOI:** 10.3390/jcm10081770

**Published:** 2021-04-19

**Authors:** Manoja P. Herath, Kiran D. K. Ahuja, Jeffrey M. Beckett, Sisitha Jayasinghe, Nuala M. Byrne, Andrew P. Hills

**Affiliations:** School of Health Sciences, College of Health and Medicine, University of Tasmania, Launceston, TAS 7248, Australia; manoja.herath@utas.edu.au (M.P.H.); kiran.ahuja@utas.edu.au (K.D.K.A.); jeffrey.beckett@utas.edu.au (J.M.B.); sisitha.jayasinghe@utas.edu.au (S.J.); nuala.byrne@utas.edu.au (N.M.B.)

**Keywords:** pre-pregnancy factors, prenatal factors, postnatal factors, infants, fat, adiposity, programming

## Abstract

Excess adiposity in infancy may predispose individuals to obesity later in life. The literature on determinants of adiposity in infants is equivocal. In this longitudinal cohort study, we investigated pre-pregnancy, prenatal and postnatal determinants of different adiposity indices in infants, i.e., fat mass (FM), percent FM (%FM), fat mass index (FMI) and log-log index (FM/FFM*^p^*), from birth to 6 months, using linear mixed-effects regression. Body composition was measured in 322, 174 and 109 infants at birth and 3 and 6 months afterwards, respectively, utilising air displacement plethysmography. Positive associations were observed between gestation length and infant FM, maternal self-reported pre-pregnancy body mass index and infant %FM, and parity and infant %FM and FMI at birth. Surprisingly, maternal intake of iron supplements during pregnancy was associated with infant FM, %FM and FMI at 3 months and FM/FFM*^p^* at 6 months. Male infant sex and formula feeding were negatively associated with all adiposity indices at 6 months. In conclusion, pre-pregnancy and pregnancy factors influence adiposity during early life, and any unfavourable impacts may be modulated postnatally via infant feeding practices. Moreover, as these associations are dependent on the adiposity indices used, it is crucial that researchers use conceptually and statistically robust approaches such as FM/FFM*^p^*.

## 1. Introduction

The prevalence of obesity and comorbidities are rising at alarming rates across all age groups worldwide [1]. Growth and development in early life play a pivotal role in determining the risk of obesity over the life course [2]. Programming of the growth trajectory in humans occurs during the first 1000 days of life (from conception to 2 years of age), when developmental plasticity is at its maximum [3]. Across this critical period, insults or inappropriate stimuli can result in metabolic and structural alterations in cells, organs and systems that may be irreversible [4]. For example, excess accumulation of adipose tissue in foetal life or early infancy may predispose individuals to obesity in later childhood and adulthood [5].

Several pre-pregnancy, prenatal and postnatal factors have been identified as predictors of adiposity in infants, including maternal pre-pregnancy body mass index (ppBMI), education, low socioeconomic status, smoking and infant feeding mode [6,7,8]. Nevertheless, the literature is equivocal on the impacts of some of the factors, and other factors have not been adequately studied. For example, considerable evidence suggests that maternal obesity during conception is associated with increased infant fat mass (FM) [9,10], but this relationship was not evident in other studies [11,12]. Similarly, the positive association between exposure to gestational diabetes mellitus (GDM) in utero and FM in newborns reported in some studies [13,14,15] was not found in others [16,17,18]. Additionally, although the use of vitamin and mineral supplementation is common in pregnancy, evidence on how these supplements affect the growth and body composition of infants is scarce [19]. Finally, maternal factors and their effects on infant adiposity have more commonly been studied at a single time-point, in most cases at birth [20,21]. As associations between maternal factors and adiposity at birth have been reported to change over the first 5 months of life [6], studying the combined effect of a range of pre-pregnancy, prenatal and postnatal factors throughout infancy could provide a better understanding of the modulation of adiposity accretion during this critical period.

Accurate estimation of adiposity in infants was challenging until the development of the PEA POD air displacement plethysmography (ADP) system, a rapid and non-invasive two-compartment technique with excellent reliability and validity [22]. The expression of the outcomes of the two-compartment model, i.e., FM and fat-free mass (FFM), as absolute values without adjusting for body size, can compromise its clinical relevance. For example, FM alone cannot elucidate interindividual variability of fatness, nor can it rank individuals in terms of disease risk [23]. Nonetheless, the absolute values of FM have been used as a measure of adiposity in infants [24]. Additionally, different indices derived from FM, including %FM (FM adjusted for total body weight) [7], FMI (FM adjusted for height/length) [25], and FM/FFM*^p^* (FM adjusted for FFM) [26], have been used, but their interrelationships and, whether their determinants are identical, have not been investigated thoroughly. 

Identifying the links between pre-pregnancy and prenatal factors and neonatal adiposity could provide valuable insights for optimising maternal health to promote better cardiometabolic outcomes later in life for their offspring. Following up on these associations across infancy, with adjustments for key confounding postnatal factors, may advance the understanding of how long these prenatal influences can last and how any undesirable impacts can be ameliorated postnatally. Moreover, comprehension of associations between these factors and various adiposity indices may inform the selection of appropriate measure(s) for future research. In this longitudinal cohort study, we investigated associations between pre-pregnancy, prenatal and postnatal factors and different measures of infant adiposity from birth to 6 months.

## 2. Materials and Methods

### 2.1. Participant Recruitment

The Baby-bod study is the Australian arm of a multi-country collaborative project exploring body composition in healthy infants across the first two years of life. Participant recruitment and all assessments in the Baby-bod study were conducted at the Launceston General Hospital, Tasmania, Australia, from September 2017 to October 2019. Inclusion criteria were: mothers with a singleton term pregnancy (gestation length at birth between 37 ^+0^ and 41 ^+6^ weeks), and ≥18 years of age and able to speak and understand English. Exclusion criteria were: newborns with congenital anomalies or admitted to the neonatal intensive care unit, mother’s inability to negotiate the informed consent process, and difficult birthing experience as judged by a clinician. All eligible mothers were approached within 72 h of delivery and provided with participant information sheets. Informed written consent was obtained from the mothers (also from the fathers, if present) who agreed to participate in the first assessment. All research procedures and protocols were approved by the Human Research Ethics Committee of Tasmania (reference: H0016117).

### 2.2. Assessment of Exposure Variables

Maternal demographic information (age, ethnicity, highest education, occupational status, parity), lifestyle characteristics (smoking, routine intake of iron and folic acid throughout pregnancy), diagnosis of GDM, and pre-pregnancy weight were obtained through an interviewer-administered screening questionnaire. Infant feeding pattern in the month prior to each follow-up visit (3 and 6 months) was obtained using a food frequency questionnaire. All questionnaires used in this study were designed for the multi-country project and used at all the study sites.

### 2.3. Anthropometric and Body Composition Assessment of Infants 

Infant weight, length, head circumference and body composition were recorded at birth and 3 and 6 months after. Nude weight was obtained using a Seca 374 digital baby scale (Seca, Hamburg, Germany) to the nearest 5 g up to 7.5 kg and the nearest 10 g beyond. Crown-to-heel length was measured using a Seca 417 infantometer (Seca, Hamburg, Germany) to the last completed millimetre. A flexible steel tape (Cescorf, Porto Alegre, Brazil) was used to assess head circumference to the last completed millimetre. All measurements were taken in duplicate by two trained research assistants and averaged. When the duplicate readings were out of the study’s tolerance ranges, i.e., weight: 50.0 g, length: 7.0 mm, and head circumference: 5 mm, the assessment was repeated, and only measurements within the tolerance were averaged. 

Body composition in infants was assessed using the PEA POD ADP system (COSMED USA Inc., Concord, CA, USA; software version 3.5.0) according to the manufacturer’s guidelines. PEA POD, a two-compartment approach, uses the gas laws of Boyle and Poisson, and principles of whole-body densitometry. Body density was used to estimate FM, assuming that the density of FM was constant and assigning age and gender-specific FFM density values determined by Fomon et al. [27]. The physical design and the operating procedures of the PEA POD have been described in detail elsewhere [22]. 

### 2.4. Calculation of Infant Adiposity Indices

FMI was calculated as FM (kg)/length (m)^2^. %FM was calculated as FM (kg) *100%/body weight (kg). For calculating FM/FFM*^p^*, first FM and FFM were natural log-transformed. Next, log FM was regressed on log FFM to find distinct regression coefficients for each time-point. These coefficients were used for p to calculate FM/FFM*^p^*, and the derived values were multiplied by 1000 to enhance the readability [26].

### 2.5. Maternal Anthropometry

Post-delivery weight was measured at enrolment (within 72 h of delivery) using a Seca 876 flat scale (Seca, Hamburg, Germany) to the nearest 0.1 kg. Height was measured using a Seca 264 digital stationary stadiometer (Seca, Hamburg, Germany) to the nearest 0.1 cm at enrolment or the first follow-up visit in cases when the mother was unable to stand after the delivery. ppBMI was calculated as the mother’s self-reported pre-pregnancy weight (kg) divided by height squared (m^2^). Maternal net gestational weight gain (nGWG) was calculated as the difference between the mother’s postdelivery weight (kg) and self-reported pre-pregnancy weight (kg).

### 2.6. Statistical Analysis

Continuous predictors were: maternal age (years), parity, ppBMI (kg/m^2^) and nGWG (kg). Categorical predictors were: diagnosis of GDM (yes vs. no (reference group)), routine intake of supplemental iron (yes vs. no (reference group)) and folic acid (yes vs. no (reference group)), smoking (0–3 days (reference group) vs. 4–7 days per week) during pregnancy, highest education (up to high school vs. university/professional training (reference group)), occupational status (unemployed vs. employed (reference group)), infant sex (male vs. female (reference group)) and infant feeding mode (exclusive breastfeeding (reference group), partial breastfeeding (both breastmilk and formula milk), and formula feeding). Longitudinal associations of predictor variables and infant adiposity indices were examined with linear mixed-effects (LME) models using backward stepwise regression. LME models take the correlation between repeated measures on the same individual into account and allow for missing data in the outcome measure, assuming that it was missing at random [28]. Moreover, LME modelling requires the presence of all the predictor variables at all the time-points considered. Consistent with the multi-country study, we collected data on infant feeding only at 3 and 6 months. Thus, separate models were developed considering all 3 time-points, i.e., birth, 3 months and 6 months (Model 1), and considering only 3 months and 6 months (Model 2). Model 1 included all pre-pregnancy and pregnancy predictor variables considered. Model 2 included all variables in Model 1 plus infant feeding mode and respective adiposity measure at birth. A *p*-value < 0.2 was considered the cut-off inclusion of a predictor variable in the model. Infant sex and gestation length were included in the models independent of their *p* values. The interactions between maternal factors and infant age at the assessment, i.e., birth (reference level), 3 months and 6 months, were explored to understand whether the associations of maternal prenatal factors changed with infant age. Variance inflation factors (VIFs) were computed to confirm there was no multicollinearity between exposure variables (all <2.0). Residual plots of each model were examined to confirm the assumptions of normality, linearity and homoscedasticity. Cook’s distance influence statistics were computed to identify unduly influential observations in the model construction, and the values flagged as being influential were checked for genuineness. All statistical analyses were conducted using R Project for Statistical Computing (version 3.5.3) in R Studio (version 1.1.463, Vienna, Austria) [29]. Statistical significance level was two-tailed and set at *p* < 0.05. 

## 3. Results

### 3.1. Participants

Of the 1375 mothers approached, 322 mothers (44.5% primiparous) agreed to participate in the study. Their newborns were assessed at birth, and 191 (58.6%) of them at-tended the 3-month follow-up, and 183 (56.1%) attended the 6-month follow-up (Supplementary Materials Figure S1). ADP measurements were available for 174 infants at 3 months and 109 infants at 6 months. Reasons cited for lack of follow-up included lack of time and moving to another area. Infants with complete data for all variables of interest were included in the regression analyses. Maternal and infant characteristics were similar for the group who commenced the study and those who were included in the final analyses (Supplementary Materials Table S1).

### 3.2. Baseline Characteristics of Mothers

The age of mothers at delivery ranged from 18 to 48 years, with a mean ± SD of 29.9 ± 5.2 years. Mothers were predominantly Caucasian (91.3%), and most routinely consumed supplemental iron (79.5%) and supplemental folic acid (64.1%) throughout pregnancy (Table 1). Of the 297 mothers who had records of ppBMI, more than half were overweight, and nearly 1 in 10 mothers had been diagnosed with GDM. There were no significant differences in maternal sociodemographic characteristics (age, ethnicity, highest education, occupational status, parity, height and pre-pregnancy weight) between the full (*n* = 322) and analytical cohorts (*n* = 235) (Supplementary Materials Table S1).

### 3.3. Infant Characteristics from Birth to 6 Months

The weight of infants increased by 80% from birth to 3 months (3283 ± 449.3 g vs. 5931 ± 801.3 g), in contrast to a 30% increase from 3 to 6 months (7632 ± 946.2 g at 6 months) (Table 2). As expected, there were significant changes in all adiposity measures; greater increases from birth to 3 months and relatively smaller increases from 3 to 6 months (all *p* < 0.001). On average, FM increased by 3-fold from birth to 3 months (353.6 ± 161.0 vs. 1412.3 ± 397.8), but the increase was smaller from 3 to 6 months (1859 ± 412.4 g at 6 months). %FM more than doubled during the first 3 months (10.5% ± 3.8% vs. 23.7% ± 4.6%); however, the increase was not as pronounced during the subsequent 3-month period (25.4% ± 4.5% at 6 months). A similar trend was observed in FMI (birth: 1.4 ± 0.6; 3 months: 3.9 ± 1.1; 6 months: 4.4 ± 1.0). Conversely, FM/FFM*^p^* displayed dramatic changes corresponding to fluctuations in FM and FFM in 0–6-month-old infants. FM/FFM*^p^* increased by approximately 6-fold during the first 3 months (36.0 ± 14.4 at birth vs. 251.5 ± 62.9 at 3 months), and by 5-fold during the next 3 months (1519 ± 336.1 at 6 months). Infant feeding mode changed substantially from 3 months (68.9% exclusively breastfed) to 6 months (16.9% exclusively breastfed).

### 3.4. Correlation between Infant Adiposity Indices

Overall, adiposity indices were positively correlated with each other. FM, %FM and FMI were highly correlated (*r* > 0.80), while their correlation with FM/FFM*^p^* was moderate (*r* = 0.65–0.78). 

### 3.5. Associations between Maternal Prenatal Factors and Infant Adiposity Indices

#### 3.5.1. FM (g)

Gestation length was positively associated with FM at birth (34.07; 0.64 to 67.48, Table 3), but this effect was not evident at 3 and 6 months (Table 4). The increase in FM was significantly smaller from birth to 3 months (−205.3; −352.30 to −58.36, Table 3) and from birth to 6 months (−273.72; −446.49 to −100.95, Table 3) in infants born to mothers who had supplemental iron during pregnancy compared to the infants of mothers who did not consume supplemental iron, and these effects did not significantly change even after adjusting the effects for the infant feeding mode (Table 4). Growth in FM from 3 to 6 months was lower in male infants compared to female infants (−73.14; −305.70 to −40.67, Table 4), and in formula-fed infants compared to exclusively breastfed infants (−248.27; −470.16 to −25.62, Table 4).

#### 3.5.2. %FM

ppBMI (0.08; 0.005 to 0.016) and parity (0.72; 0.21 to 1.24, Table 3) were positively associated with %FM at birth, and these effects did not last at 3 or 6 months. The association between iron intake and %FM was not significant at birth; however, in infants born to mothers who consumed iron supplements, the increase in %FM was significantly lower at 3 months (−2.47; −4.24 to −0.70, Table 3) and 6 months (−2.95; −5.06 to −0.83, Table 3) compared to infants of mothers who did not take iron supplements, and similar effects were found after accounting for the feeding mode (Table 4). Increases in %FM from 3 to 6 months were lesser in male infants compared to female infants (−2.14; −3.75 to −0.54, Table 4), and in formula-fed infants compared to exclusively breastfed infants (−3.84; −6.47 to −1.21, Table 4).

#### 3.5.3. FMI

Parity was the only significant predictor of FMI at birth (0.12; 0.02 to 0.22, Table 3). Compared to infants of mothers who did not take iron supplements, the increase in FMI was significantly lower in infants born to mothers who had supplemental iron during pregnancy from birth to 3 months (−0.48; −0.82 to −0.13, Table 3) and from birth to 6 months (−0.53; −0.95 to −0.12, Table 3), which did not significantly change after adjusting for the infant feeding mode. Elevations in FMI from 3 to 6 months were smaller in male infants compared to female infants (−0.47; −0.78 to −0.16, Table 4), and in formula-fed infants compared to exclusively breastfed infants (−0.81; −1.33 to −0.29, Table 4).

#### 3.5.4. FM/FFM*^p^*

None of the potential predictors considered in our analysis were associated with FM/FFM*^p^* at birth. The increase in FM/FFM*^p^* from birth to 6 months was lower in infants born to mothers who had iron supplements (−216.22; −310.57 to −121.89, Table 3) and higher in those of mothers who had folic acid supplements (112.53; 31.03 to 194.05, Table 3); however, only the effect of iron was significant when adjusted for the effect of infant feeding. Similar to all other indices, increases in FM/FFM*^p^* from 3 to 6 months were also lower in male infants (−119.08; −225.99 to −12.80, Table 4) and formula-fed infants (−162.75; −322.41 to −4.23, Table 4).

## 4. Discussion

In this longitudinal cohort study, we explored associations between pre-pregnancy, prenatal and postnatal factors and infant adiposity measured with four indices—namely, FM, %FM, FMI and FM/FFM*^p^*, across 0–6 months of age. We identified positive associations between gestation length and infant FM, maternal self-reported ppBMI and infant %FM, and parity and infant %FM and FMI, at birth. Surprisingly, maternal intake of iron supplements during pregnancy was negatively associated with infant FM, %FM and FMI at 3 months, and FM/FFM*^p^* at 6 months. Male infant sex and formula feeding were negatively associated with all adiposity indices at 6 months. Our findings imply that pre-pregnancy and pregnancy factors influence adiposity during early life, and any unfavourable impacts may be modulated during the postnatal period, particularly via infant feeding practices. Moreover, the associations we observed were dependent on the adiposity measure used. Therefore, it is critical that researchers understand the strengths and limitations of different adiposity indices and use conceptually and statistically robust approaches such as FM/FFM*^p^*.

Gestation length at delivery has been reported as the strongest predictor of birth measurements in many studies. Positive associations of gestation length with infant FM found in the current study is in accordance with the earlier findings [6,30,31,32]. Moreover, we observed a positive effect of parity on infant %FM and FMI at birth. Increases in infant fatness in successive pregnancies have been explained as a function of changes in the mother’s metabolism as a cumulative effect of advancing age and prior pregnancies [33]. 

Furthermore, ppBMI is an indicator of maternal nutrition status during conception. The increases in newborn adiposity with increasing ppBMI has been explained by the fact that high blood glucose levels in mothers with excess weight triggers the production of insulin that in turn increases lipogenesis and excessive fat deposition in the foetus [34]. Our findings of the impact of ppBMI were consistent with those who reported a positive association between ppBMI and %FM at birth [7,35] and others who did not find significant associations at 3 months [36], 5 months [6] and 6 months [36]. However, the positive association between ppBMI and FM/FFM*^p^* at birth, showed by Abreu et al. [26], was not evident in our study. Conversely, some researchers have shown that ppBMI is not associated with FM or %FM, even in newborns [37]. 

Between 3 and 6 months, to our surprise, we found a negative association between maternal supplemental iron intake during pregnancy and infant adiposity measures, which did not significantly change even after the models were adjusted for infant feeding mode. This result may be because mothers who took iron supplements were generally more aware of health issues in pregnancy and thus led a healthier lifestyle which promoted leanness in their infants. Physiologically, it is also possible that high iron stores in infants born to mothers who took iron supplements promoted the production of red blood cells, myoglobin, and muscle growth [38], leading to the relative increase in FFM and reductions in adiposity. Further, we observed that the increase in FM/FFM*^p^* from birth to 6 months was significantly larger in infants born to mothers who consumed folic acid supplements during pregnancy, but this relationship was no longer significant after adjusting for the feeding mode. Dahly et al. [24] have also shown that FM was not different in newborns whose mothers met the recommended daily allowance of folate (400 µg dietary folate equivalents) vs. those of mothers who did not. Since our data were limited to whether mothers consumed supplemental iron/folic acid during pregnancy or not, our results should be interpreted with caution. Future research should consider the dosage and length of supplement intake during pregnancy on infant adiposity at birth and long-term.

Another predictor of adiposity increase from 3 to 6 months of age was infant sex. Prior studies have also noted the higher adiposity levels in female infants in contrast to higher FFM in male infants [6,21,32,36]. These differences have been explained by higher levels of testosterone produced by the testes in the male infants, which promote the growth in FFM [39] and higher concentrations of plasma leptin (a known regulator of appetite) in female infants, stimulating larger intakes of milk, which leads to deposition of energy as fat [40]. Furthermore, compared to infants who were exclusively breastfed, formula-fed infants had a significantly lower increase in adiposity (evident in all indices) from 3 to 6 months. Our result is consistent with the findings of a systematic review of 15 studies that compared the body composition of breastfed vs. formula-fed infants [41]. The authors reported that compared to breastfed infants, formula-fed infants had lower FM and %FM at 3–4 months, as well as at 6 months, and this could be due to higher leptin levels, characteristic of breastfed infants. Another possibility would be that formula milk contains more energy and protein compared to breastmilk, which may promote growth in FFM, thereby resulting in lower levels of relative fatness [42]. However, in our infants, this difference in adiposity attributed to the feeding mode was not evident at 3 months. The reason could be that we analysed infants’ feeding mode with 1-month feed recall, and it may have not accurately depicted the infants’ diet in the period of 0–3 months. On the other hand, the reason may be that more time was needed to reflect the changes due to differences in feeding mode in infants’ body compositions. Nevertheless, despite the increased adiposity found in breastfed infants compared to formula-fed infants during 0–6 months of age, a large body of evidence suggests breastfeeding has a protective effect against obesity and adiposity later in life [43,44].

Previous studies have identified excessive gestational weight gain (GWG) during pregnancy based on the weight gained between conception and the onset of labour, following the recommendations of the Institute of Medicine. This includes the weight of the infant, placenta and amniotic fluid, which account for ~35% of the GWG; therefore, it may not accurately reflect the actual weight gain of the mother [32]. In our analysis, we used nGWG (weight calculated as the difference between pre-pregnancy and post-labour weight) to assess the association between true weight gain of the mother and measures of infant adiposity and did not find any association. Except for one study [45] that investigated the association between nGWG and risk of large-for-gestational-age birth, nGWG has not been considered as a predictor of infant adiposity in any previous studies, and it is, therefore, difficult to compare our results. If nGWG is adopted in future research, it may help to identify more accurate associations specific to the real weight gain of the mother. Moreover, GDM was not a significant predictor of adiposity in our infants; however, we acknowledge the low number of mothers with GDM in our study precludes a robust conclusion. In our systematic review and meta-analysis of studies comparing adiposity in infants born to mothers with GDM and mothers with normal glucose tolerance, we have shown that, despite treatments for GDM, infants exposed to GDM in utero had higher total body adiposity than the infants born to mothers with normal glucose tolerance [34]. Infants of mothers who smoked during pregnancy are distinguished by lower FFM [31,46], which might affect measures of %FM or FM/FFM*^p^*. Nonetheless, we did not observe a significant relationship between maternal smoking and infant adiposity, potentially due to the nature of the approach we used in our data collection. The smoking status of mothers was recorded as a dichotomous variable as “smoked 0–3 days” or “smoked 4–7 days”; hence, (light) smokers were included in the same group as non-smokers. Further, our maternal cohort was predominantly Caucasian, employed and had university education/professional training. Consequently, maternal ethnicity, occupation status, and education were not significant predictors of infant adiposity.

Although adiposity is widely expressed with absolute values of FM, normalising FM for size is fundamental to understand the relative fatness of individuals [23]. Most commonly, FM is normalised for overall body weight, i.e., %FM, but this approach cannot effectively distinguish fatness between individuals as %FM is affected by changes in FM as well as FFM [47]. FMI is recommended as a more appropriate approach that allows independent evaluation of FM relative to body size (height) [23]. In addition, recent research has demonstrated that FMI is a more reliable index than %FM when assessing neonatal adiposity [48]. In contrast, some studies [26,47] have suggested that an appropriate index should adjust the originator of the risk (FM) for a variable that is bearing the risk (FFM), thus recommending the index of FM/FFM*^p^*; however, to the best to our knowledge, only one study [26] used FM/FFM*^p^* to identify maternal predictors of infant adiposity at birth. Our data demonstrate that FM/FFM*^p^* is highly sensitive to rapid changes in adiposity during this critical period of growth, with drastic increases from birth (~36) to 6 months (~1500). Further research is required to test its reliability and validity in longitudinal studies. 

To our knowledge, this is the first study that concurrently explores the determinants of different indices of adiposity in early infancy. Other strengths of our study are the use of a validated and reliable technique to evaluate infant body composition, prospective longitudinal study design and satisfactory sample size compared with similar studies. Nonetheless, the inclusion of mostly healthy mother-infant dyads and participant loss-to-follow-up may have introduced selection bias to our study and limits the generalisability of our findings. Despite the PEA POD being the most “practical” body composition assessment technique for infants of 1–10 kg body weight, potential sources of measurement error include hydration status, body moisture, temperature, and hair [49]. Another limitation of our study is that the maternal variables, including pre-pregnancy weight, were self-reported by mothers in an interview-based approach and can be subjected to recall inaccuracies and social desirability bias. Particularly, self-reported ppBMI is used in many studies as a crude measure of maternal adiposity as obtaining objective maternal body composition measurements before conception and throughout pregnancy would be extremely difficult in practice, although it would be ideal. We also concede that the associations we report on prenatal supplements have been analysed without considering the variations in doses; therefore, the results should be translated with caution. Further, we acknowledge there are other potential predictors of neonatal body composition that we have not adjusted for in our results. These may include prenatal factors such as maternal dietary intake [50], use of other micronutrient supplements (e.g., vitamin D [51], iodine [52]) or combined nutritional supplements during pregnancy [53], maternal physical activity level [54], and postnatal factors such as infant milk feeding patterns (e.g., variations in volume and frequency [55]) and infants’ exposure to micronutrients (e.g., iron supplementation is recommended for breastfed infants since the concentration of iron in breastmilk is very low and declines with time; in contrast, formula-fed infants may get iron from iron-fortified formula [38]). 

## 5. Conclusions

We identified that gestation length, parity, and ppBMI were significant predictors of adiposity in newborns, and male infant sex, intake of supplemental iron during pregnancy, and mode of feeding significantly contributed to variations in adiposity of 3–6-month-old infants. However, these associations were dependent on the adiposity index used. Our results highlight the importance of optimal maternal health and lifestyle during pre-pregnancy and pregnancy periods in determining adiposity in early life. Our findings also suggest that any negative prenatal impacts on neonatal adiposity may be ameliorated during the postnatal period, potentially with infant feeding practices. Additionally, it is critical that researchers understand the strengths and limitations of respective approaches when choosing a measure of adiposity to investigate its relationship with potential predictor variables. On the understanding that FM cannot identify relative fatness of individuals and %FM is statistically flawed, future research should use conceptually and statistically more robust adiposity measures. FM/FFM*^p^* can account for changes in both FM and FFM; therefore, we suggest that it may be a better index for tracking variations in relative fatness in infants and identifying relationships with maternal factors. Further, longitudinal studies beyond early infancy are required to inform long-standing links between pre-pregnancy, prenatal and postnatal factors and offspring growth trajectory.

## Figures and Tables

**Table 1:** Baseline characteristics of mothers.

Variable (*n* = 322)	Missing Values	*n* (%)/Mean (± SD)/ Median (25th and 75th Percentiles)
Age (years) *	0	29.9 (5.2)
Ethnicity	1	
Caucasian		294 (91.3)
Other		27 (8.4)
Highest education	0	
University/professional training		235 (73.0)
Up to high school		87 (27.0)
Occupational status	0	
Employed		283 (87.9)
Unemployed		39 (12.1)
Parity	1	
Primiparous		143 (44.5)
Multiparous		178 (55.5)
Pre-pregnancy BMI (kg/m^2^) **	25	25.2 (22.0, 29.7)
Pre-pregnancy weight status	25	
Non-overweight (BMI < 25)		142 (47.8)
Overweight (BMI ≥ 25)		155 (52.2)
Net gestational weight gain (nGWG) **	8	8.5 (4.2, 12.4)
Gestational diabetes	0	
No		290 (90.1)
Yes		32 (9.9)
Smoking during pregnancy	0	
0–3 days per week		304 (94.4)
4–7 days per week		18 (5.6)
Intake of supplemental iron	30	
Yes		232 (79.5)
No		60 (20.5)
Intake of supplemental folic acid	46	
Yes		177 (64.1)
No		99 (35.9)
Gestation length (weeks) *	0	39.5 (1.1)

Numbers represent count (%) for categorical variables and mean (SD) *, or median (25th and 75th percentiles) ** for continuous variables; SD: standard deviation; BMI: body mass index; nGWG: net gestational weight gain.

**Table 2:** Characteristics of infants at birth, 3 months and 6 months.

Variable	Birth		3 Months		6 Months	
	*n*	Mean (SD)	*n*	Mean (SD)	*n*	Mean (SD)
Age (days)	317	1.8 (1.1)	191	88.3 (7.9)	181	180.3 (8.4)
Sex *	322		191		182	
Female		167 (51.9)		100 (52.4)		93 (51.1)
Male		155 (48.1)		91 (47.6)		89 (48.9)
Weight (g)	317	3283 (449.3)	191	5931 (801.3)	181	7632 (946.2)
Length (cm)	316	49.5 (2.1)	191	59.8 (2.2)	182	66.1 (2.5)
Head circumference (cm)	317	34.4 (1.2)	191	40.2 (1.2)	180	43.1 (1.3)
FM (g)	314	353.6 (161.0)	174	1412.3 (397.8)	109	1859 (412.4)
FFM (g)	314	2933 (346.7)	174	4473 (477.0)	109	5421 (557.1)
%FM	314	10.5 (3.8)	174	23.7 (4.6)	109	25.4 (4.5)
%FFM	314	89.5 (3.8)	174	76.3 (4.6)	109	74.6 (4.5)
FMI (kg/m^2^)	314	1.4 (0.6)	174	3.9 (1.1)	109	4.4 (1.0)
FFMI (kg/m^2^)	314	12.0 (0.9)	174	12.5 (0.9)	109	12.6 (0.8)
FM/FFM*^p^*	314	36.0 (14.4)	174	251.5 (62.9)	109	1519 (336.1)

Numbers represent mean (SD) for continuous variables and count (%) for categorical variables *; SD: standard deviation; n: number of infants for each variable of interest; FM: fat mass; FFM: fat-free mass; %FM: percent fat mass; %FFM: percent fat-free mass; FMI: fat mass index; FFMI: fat-free mass index; *p*: relevant regression coefficient (described in detail in methods).

**Table 3:** Longitudinal associations between pre-pregnancy and prenatal factors and indices of infant adiposity from birth to 6 months.

Parameter	FM (g)		%FM		FMI		FM/FFM*^p^*	
	Estimate	95% CI	Estimate	95% CI	Estimate	95% CI	Estimate	95% CI
ppBMI (kg/m^2^)							NS	
Association at birth	2.31	(−3.20 to 7.83)	0.08	(0.005 to 0.016)	0.01	(−0.002 to 0.03)		
Change from birth to 3 months	4.95	(−3.14 to 13.03)	0.02	(−0.09 to 0.014)	0.01	(−0.02 to 0.03)		
Change from birth to 6 months	−1.46	(−11.45 to 8.53)	0.09	(0.23 to 0.06)	−0.01	(−0.04 to 0.02)		
nGWG (kg)	NS				NS		NS	
Association at birth			0.04	(−0.02 to 0.05)				
Change from birth to 3 months			0.004	(−0.04 to 0.05)				
Change from birth to 6 months			−0.002	(−0.06 to 0.05)				
Gestational diabetes: yes	NS		NS				NS	
Association at birth					−0.17	(−0.52 to 0.18)		
Change from birth to 3 months					−0.27	(−0.80 to 0.27)		
Change from birth to 6 months					0.1	(−0.47 to 0.68)		
Intake of supplemental iron: yes								
Association at birth	17.01	(−85.27 to 119.34)	0.42	(−0.80 to 1.64)	0.07	(−0.17 to 0.31)	2.45	(−49.26 to 54.16)
Change from birth to 3 months	**−205.34**	**(−352.30 to −58.36)**	**−2.47**	**(−4.24 to −0.70)**	**−0.48**	**(−0.82 to −0.13)**	−36.53	(−118.00 to 44.86)
Change from birth to 6 months	**−273.72**	**(−446.49 to −100.95)**	**−2.95**	**(−5.06 to −0.83)**	**−0.53**	**(−0.95 to −0.12)**	**−216.22**	**(−310.57 to −121.89)**
Intake of supplemental folic acid: yes			NS		NS			
Association at birth	−4.54	(−92.84 to 83.81)					−0.88	(−45.42 to 43.67)
Change from birth to 3 months	101.77	(−27.38 to 230.93)					12.01	(−59.24 to 83.38)
Change from birth to 6 months	142.11	(−6.84 to 291.07)					112.53	(31.03 to 194.05)
Parity								
Association at birth	29.72	(−8.90 to 68.33)	**0.72**	**(0.21 to 1.24)**	**0.12**	**(0.02 to 0.22)**	2.57	(−16.94 to 22.08)
Change from birth to 3 months	−20.8	(−79.28 to 37.69)	−0.51	(−1.31 to 0.30)	−0.12	(−0.28 to 0.05)	0.62	(−59.24 to 83.38)
Change from birth to 6 months	22.62	(−42.67 to 37.69)	−0.09	(−1.00 to 0.80)	0.01	(−0.17 to 0.19)	46.87	(31.03 to 194.05)
Gestation length (weeks)								
Association at birth	**34.07**	**(0.64 to 67.48)**	0.44	(−0.01 to 0.89)	0.08	(−0.01 to 0.18)	−0.52	(−17.38 to 16.34)
Change from birth to 3 months	−13.53	(−62.01 to 34.93)	−0.69	(−1.36 to −0.02)	−0.13	(−0.27 to 0.002)	−6.03	(−32.70 to 20.61)
Change from birth to 6 months	5.96	(−50.74 to 34.93)	−0.38	(−1.18 to 0.41)	−0.05	(−0.21 to 0.10)	34.33	(3.48 to 65.18)
Infant sex: male								
Association at birth	−6.14	(−82.30 to 70.11)	−0.71	(−1.73 to 0.32)	−0.10	(−0.30 to 0.11)	−5.69	(−44.23 to 32.85)
Change from birth to 3 months	39.57	(−69.50 to 148.64)	−0.66	(−2.17 to 0.86)	−0.04	(−0.33 to 0.26)	−19.82	(−80.11 to 40.51)
Change from birth to 6 months	**−145.16**	**(−273.16 to −17.15)**	**−2.74**	**(−4.50 to 0.97)**	**−0.52**	**(−0.87 to −0.17)**	**−141.1**	**(−211.12 to −71.11)**

Estimates of the predictors were obtained from Model 1 of stepwise mixed-effects linear regression conducted with backward elimination (at *p* > 0.2) separately for each outcome measure. Gestation length and infant sex were included in all the models; FM: fat mass; FFM: fat-free mass; %FM: percent fat mass; %FFM: percent fat-free mass; FMI: fat mass index; ppBMI: pre-pregnancy body mass index; nGWG: net gestational weight gain. Reference groups for categorical variables include gestational diabetes: no, antenatal iron: no, antenatal folic acid: no, infant sex: female. NS indicates that the predictor was removed from the model during backward elimination. Other exposure variables and covariates considered and removed due to statistical non-significance and not shown in the table include maternal age, antenatal smoking, antenatal and interaction between ppBMI and nGWG. Interaction effects at 3 and 6 months show the change in the association from birth to 3 months and change in the association from birth to 6 months, respectively. Bold values denote statistical significance at *p* < 0.05.

**Table 4:** Longitudinal associations between pre-pregnancy, prenatal and postnatal factors and indices of infant adiposity from 3 to 6 months.

Parameter	FM (g)		%FM		FMI		FM/FFM*^p^*	
	Estimate	95% CI	Estimate	95% CI	Estimate	95% CI	Estimate	95% CI
ppBMI (kg/m^2^)							NS	
Association at 3 months	4.22	−5.46 to 13.95	0.12	−0.01 to 0.24	0.01	−0.01 to 0.04		
Change from 3 to 6 months	−3.99	−14.80 to 6.81	−0.09	−2.33 to 0.05	−0.01	−0.03 to 0.02		
nGWG (kg)	NS						NS	
Association at 3 months			0.05	−0.01 to 0.11				
Change from 3 to 6 months			−0.04	−0.09 to 0.02				
Gestational diabetes: yes	NS		NS				NS	
Association at 3 months					−0.38	−0.97 to 0.20		
Change from 3 to 6 months					0.19	−0.33 to 0.71		
Intake of supplemental iron: yes								
Association at 3 months	**−200.29**	**−370.56 to −29.53**	**−2.16**	**−3.80 to −0.53**	**−0.44**	**−0.82 to −0.06**	−41.61	−133.64 to 50.43
Change from 3 to 6 months	−79.16	−257.60 to 99.28	−0.38	−2.25 to 1.50	-0.01	−0.36 to 0.36	**−187.87**	**−329.58 to −47.13**
Intake of supplemental folic acid: yes			NS		NS			
Association at 3 months	69.53	−80.18 to 218.01					9.65	−71.74 to 91.02
Change from 3 to 6 months	57.62	−100.83 to 216.8					94.56	−29.44 to 218.09
Parity								
Association at 3 months	−5.37	−77.22 to 66.42	0.24	−0.54 to 1.02	−0.01	−0.20 to 0.18	−2.42	−40.54 to 35.69
Change from 3 to 6 months	37.64	−30.34 to 105.66	0.39	−0.42 to 1.21	0.10	−0.06 to 0.26	51.28	−3.86 to 106.43
Gestation length (weeks)								
Association at 3 months	20.40	−37.62 to 78.55	−0.31	−0.96 to 0.33	−0.02	−0.18 to 0.13	−1.13	−32.41 to 30.16
Change from 3 to 6 months	−13.50	−71.91 to 45.07	0.11	−0.61 to 0.83	−0.03	−0.17 to 0.11	38.23	−8.20 to 84.88
Infant sex: male								
Association at 3 months	31.78	−97.63 to 160.13	−1.22	−2.66 to 0.20	−0.11	−0.44 to 0.22	−16.08	−86.54 to 54.35
Change from 3 to 6 months	**−173.14**	**−305.70 to -40.67**	**−2.14**	**−3.75 to −0.54**	**−0.47**	**−0.78 to −0.16**	**−119.08**	**−225.99 to −12.80**
Feeding mode: mixed-feeding								
Association at 3 months	−142.69	−313.19 to 27.45	−1.27	−3.24 to 0.70	−0.37	−0.78 to 0.04	−20.77	−128.22 to 86.63
Change from 3 to 6 months	78.70	−152.69 to 311.11	0.53	−2.14 to 3.22	0.24	−0.30 to 0.78	−56.75	−213.94 to 99.41
Feeding mode: formula feeding								
Association at 3 months	97.99	−66.17 to 259.38	1.54	−2.90 to 3.38	0.42	−0.02 to 0.82	1.36	−92.08 to 94.81
Change from 3 to 6 months	**−248.27**	**−470.16 to -25.62**	**−3.84**	**−6.47 to -1.21**	**−0.81**	**−1.33 to −0.29**	**−162.75**	**−322.41 to −4.23**

Estimates of the predictors were obtained from Model 2 of stepwise mixed-effects linear regression conducted with backward elimination (at *p* > 0.2) separately for each outcome measure. Gestation length and infant sex were included in all the models despite their P values, and effects are adjusted for respective adiposity measure at birth; FM: fat mass; FFM: fat-free mass; %FM: percent fat mass; %FFM: percent fat-free mass; FMI: fat mass index; ppBMI: pre-pregnancy body mass index; nGWG: net gestational weight gain. Reference groups for categorical variables include gestational diabetes: no, antenatal iron: no, antenatal folic acid: no, infant sex: female, feeding mode: exclusive breastfeeding. NS indicates that the predictor was removed from the model during backward elimination. Other exposure variables and covariates considered and removed due to statistical non-significance and not shown in the table include maternal age, antenatal smoking, antenatal and interaction between ppBMI and nGWG. Interaction effects at 3 and 6 months show the change in the association from birth to 3 months and change in the association from birth to 6 months, respectively. Bold values denote statistical significance at *p* < 0.05.

## Supplementary Materials

The following are available online at https://www.mdpi.com/article/10.3390/jcm10081770/s1, Figure S1: The flow of the participants of the Baby-bod study, Table S1: Characteristics of the full cohort and analytical cohort.

## Abbreviations

FM	fat mass
%FM	percent fat mass
FMI	fat mass index
FM/FFM*^p^*	log-log index between fat mass and fat-free mass
ADP	air displacement plethysmography
ppBMI	pre-pregnancy body mass index
GDM	gestational diabetes mellitus
GWG	gestational weight gain
nGWG	net gestational weight gain
