# Supplementary material for: Determinants of Infant Adiposity across the First 6 Months of Life: Evidence from the Baby-bod study
Source: J Clin Med. 2021 Apr 19;10(8):1770. doi: 10.3390/jcm10081770 (PMC8073882; doi:10.3390/jcm10081770)
Supplement: Supplementary file 1 [file jcm-10-01770-s001.zip › jcm-1162896-supplementary.pdf]

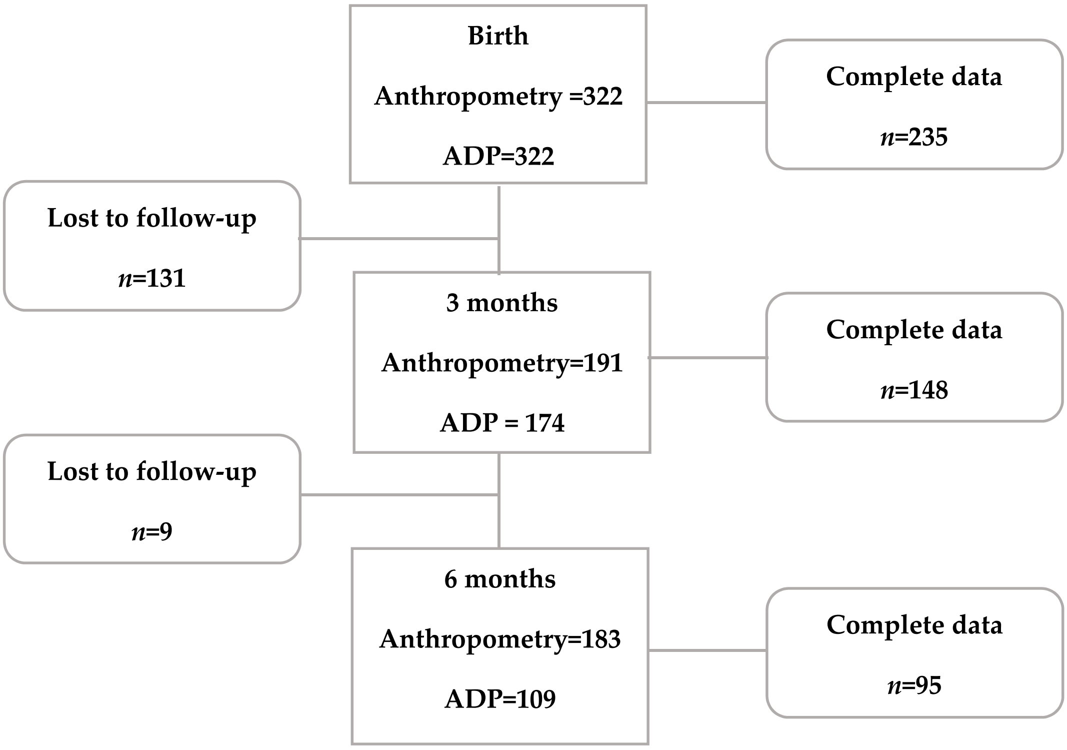

Figure S1. The flow of the participants of the Baby-bod study.

ADP: Air Displacement Plethysmography. Anthropometric measurements include weight, length, head circumference of infants. Complete data indicates the number of infants incl8uded in the analysis after removing the infants with missing data for maternal variables considered.

Table S1: Characteristics of the full-cohort and analytical cohort

Characteristic	Full cohort	Analytical cohort			
	Birth	Birth	3 months	6 months	
	N = 322	N = 235	N = 148	N = 95	*p*-value
Infant sex[1]					>0.9
Female	167 (51.9%)	127 (54.0%)	79 (53.4%)	52 (54.7%)	
Male	155 (48.1%)	108 (46.0%)	69 (46.6%)	43 (45.3%)	
Gestation length (weeks)[2]	39.50 (1.14)	39.53 (1.15)	39.61 (1.14)	39.75 (1.16)	0.3
Maternal ethnicity[1]					0.8
Caucasian	294 (91.6%)	217 (92.3%)	139 (93.9%)	89 (93.7%)	
Other	27 (8.4%)	18 (7.7%)	9 (6.1%)	6 (6.3%)	
Unknown	1				
Maternal age[2]	29.87 (5.21)	29.84 (5.26)	30.42 (5.00)	30.63 (4.56)	0.3
Parity[1]					0.9
Primiparous	143 (44.4%)	107 (45.5%)	71 (48.0%)	44 (46.3%)	
Multiparous	179 (55.6%)	128 (54.5%)	77 (52.0%)	51 (53.7%)	
Maternal prenatal BMI (kg/m^2)[3]	25.20 (22.00, 29.70	25.10 (22.30, 29.80)	25.30 (22.40, 28.85)	25.30 (22.15, 28.40)	0.7
Unknown	25				
Supplemental iron intake during pregnancy[1]					
Yes	232 (79.5%)	180 (76.6%)	112 (75.7%)	73 (76.8%)	>0.9
No	60 (20.5%)	55 (23.4%)	36 (24.3%)	22 (23.2%)	
Unknown	30				
Supplemental folic acid intake during pregnancy[1]					
Yes	177 (64.1%)	141 (60.0%)	93 (62.8%)	60 (63.2%)	0.8
No	99 (30.7%)	94 (40.0%)	55 (37.2%)	35 (36.8%)	
Unknown	46				

[1] n (%); [2]Mean (SD);[3]Median (IQR); significance tests: Pearson's Chi-squared test for categorical variables; Kruskal-Wallis rank-sum test for continuous variables; BMI: body mass index
